# Crystal structure of tris­[4-(naphthalen-1-yl)phen­yl]amine

**DOI:** 10.1107/S2056989020012529

**Published:** 2020-09-18

**Authors:** Masafumi Yano, Yukiyasu Kashiwagi, Yoshinori Inada, Yuki Hayashi, Koichi Mitsudo, Koji Kubono

**Affiliations:** a Kansai University, 3-3-35 Yamate-cho, Suita, Osaka 564-8680, Japan; b Osaka Research Institute of Industrial Science and Technology, 1-6-50 Morinomiya, Joto-ku, Osaka 536-8553, Japan; c Okayama University, 3-1-1 Tsushima-naka, Kita-ku, Okayama 700-8530, Japan; d Osaka Kyoiku University, 4-698-1 Asahigaoka, Kashiwara, Osaka 582-8582, Japan

**Keywords:** crystal structure, tri­aryl­amine, hole transporter, organic electronics, electroluminescence

## Abstract

In the title mol­ecule, C_48_H_33_N, the central N atom shows no pyramidalization, so that the N atom and the three C atoms bound to the N atom lie almost in the same plane. The three *para*-phenyl­ene rings bonded to the N atom are in a propeller form. All of the naphthalene ring systems are slightly bent. In the crystal, mol­ecules form an inversion dimer, through two pairs of C—H⋯*π* inter­actions, which further inter­acts with the adjacent dimer *via* another two pairs of C—H⋯*π* inter­actions, forming a column structure along the *a* axis. There are no significant inter­actions between these column structures.

## Abstract 2

In the crystal, two mol­ecules of the title compound form an inversion dimer, through C—H⋯*π* inter­actions, which further inter­acts with adjacent dimers to form a one-dimensional column structure.

## Chemical context   

Tri­aryl­amines (TAAs) having various substituents at their *para*-positions are widely known to give the corresponding stable cation radicals upon chemical or electrochemical one electron oxidation (Seo *et al.*, 1966 ▸). *π*-Extended TAAs with extra aromatic rings at the periphery have received considerable attention as key components in the fields of organic electroluminescence devices. Among them, the title compound was first synthesized by Kwon *et al.* (2010 ▸) as a hole-transporting material in organic light-emitting diodes. Recently, phospho­rescent organic light-emitting diodes were also reported by using the title compound as the hole-transporting material (Krucaite *et al.*, 2019 ▸). Until now, no crystal structure of this compound has been reported. We report herein the crystal structure of the title compound.
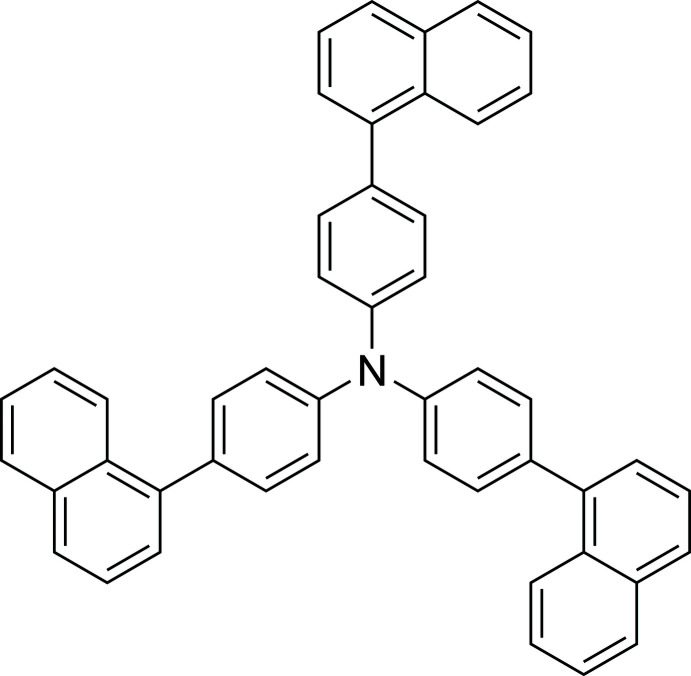


## Structural commentary   

**Figure 1:**
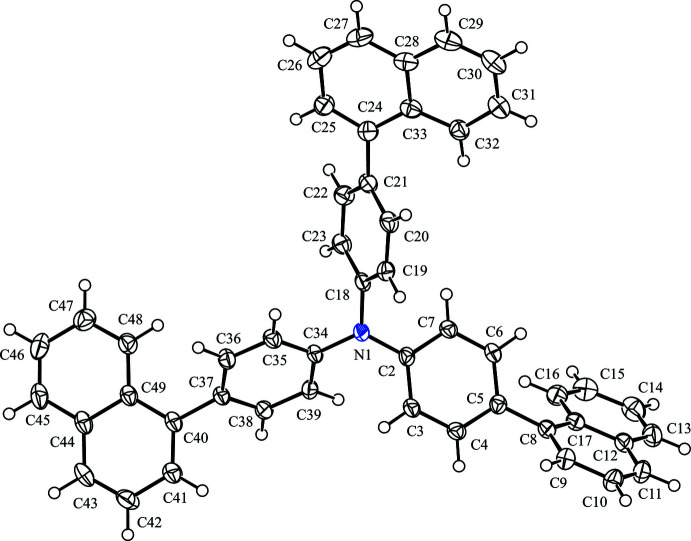
The mol­ecular structure of the title compound, with atom labeling. Displacement ellipsoids are drawn at the 50% probability level. H atoms are represented by spheres of arbitrary radius.

The mol­ecular structure of the title compound is shown in Fig. 1 ▸. The three naphthalene ring systems are slightly bent, with r.m.s. deviations of 0.038 (2), 0.055 (2) and 0.044 (2) Å, respectively, for the C8–C17, C24–C33 and C40–C49 ring systems. The C atoms at the 1-, 3- and 7-positions show the largest deviations from the mean planes [1-positions: −0.0464 (16) Å for C8, 0.0766 (18) Å for C24, and 0.0518 (17) Å for C40; 3-positions: 0.0468 (19) Å for C10, −0.068 (2) Å for C26, and −0.056 (2) Å for C42; 7-positions: 0.041 (2) Å for C15, −0.067 (2) Å for C31, and −0.051 (2) Å for C47]. In all cases, the C atoms at the 3- and 7-positions deviate from the mean plane to the same side, while the C atoms at 1-positions deviate to the opposite side. The central N1 atom shows no pyramidalization, with a deviation from the plane of the bonded C atoms (C2, C18 and C34) of 0.0402 (14) Å. The three *para*-phenyl­ene rings are bonded to the N atom in propeller-wise, which is a common arrangement for Ph_3_N fragments. The torsion angles C3—C2—N1—C34, C19—C18—N1—C2 and C35—C34—N1—C18 are −35.0 (2), −60.6 (2) and −30.3 (2)°, respectively. The *para*-phenyl­ene ring and the mean plane of the neighboring naphthalene ring system are inclined to each other by 54.66 (7)° for (C2–C7)/(C8–C17), 48.80 (7)° for (C18–C23)/(C24–C33) and 56.21 (7)° for (C34–C39)/(C40–C49).

## Supra­molecular features   

**Figure 2:**
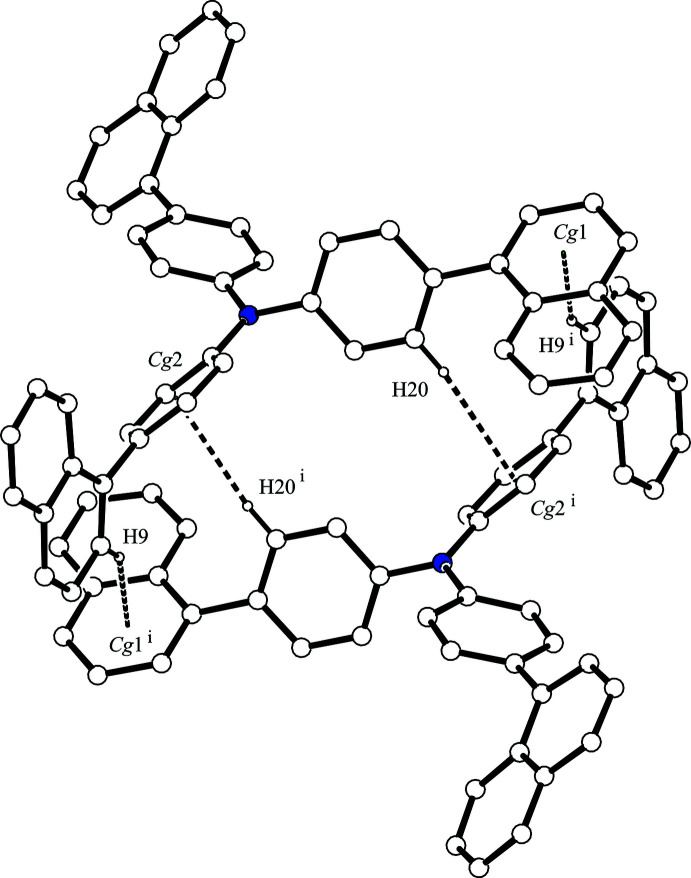
A centrosymmetric dimer of the title compound. The C—H⋯*π* inter­actions are shown as dashed lines. H atoms not involved in the inter­actions have been omitted for clarity. [Symmetry code: (i) −*x* + 1, −*y*, −*z* + 2.]

**Figure 3:**
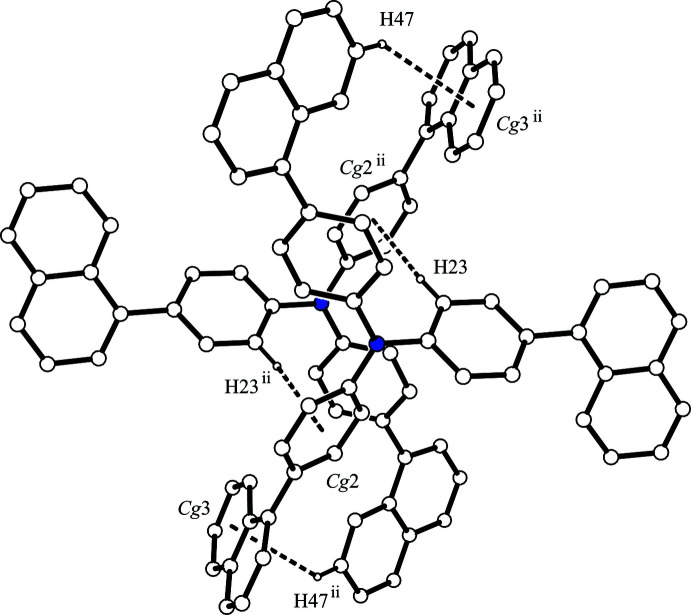
Another centrosymmetric dimer of the title compound. The C—H⋯*π* inter­actions are shown as dashed lines. H atoms not involved in the inter­actions have been omitted for clarity. [Symmetry code: (ii) −*x*, −*y*, −*z* + 2.]

**Figure 4:**
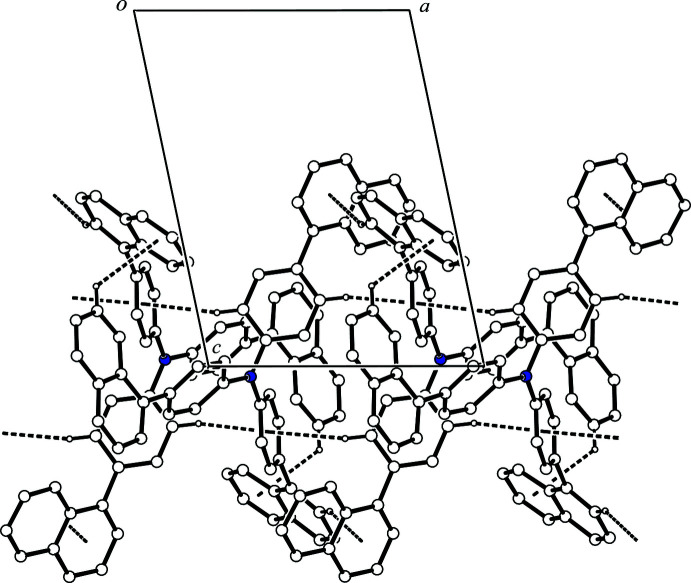
A packing diagram of the title compound viewed along the *b* axis, showing the column structure. The C—H⋯*π* inter­actions are shown as dashed lines. H atoms not involved in these inter­actions have been omitted for clarity.

In the crystal, each mol­ecule inter­acts with two others *via* four inter­molecular C—H⋯*π* inter­actions (Table 1 ▸). The mol­ecules are linked by complementary C—H⋯*π* inter­actions [C9—H9⋯*Cg*1^i^ and C20—H20⋯*Cg*2^i^; *Cg*1 and *Cg*2 are the centroids of the C24–C28/C33 and C2–C7 rings, respectively; symmetry code: (i) −*x* + 1, −*y*, −*z* + 2], forming an inversion dimer (Fig. 2 ▸). The other inversion dimer is formed by complementary C—H⋯*π* inter­actions [C23—H23⋯*Cg*2^ii^ and C47—H47⋯*Cg*3^ii^; *Cg*3 is the centroid of the C12–C17 ring; symmetry code: (ii) −*x*, −*y*, −*z* + 2] (Fig. 3 ▸). As a result, the mol­ecules form a column structure by inter­molecular C—H⋯*π* inter­actions along [100], and there is no significant inter­action between the column structures (Fig. 4 ▸).

## Database survey   

A search of the Cambridge Structural Database (CSD, Version 5.41, update August 2020; Groom *et al.*, 2016 ▸) for compounds containing tri­phenyl­amines yielded 4384 hits (including 3640 hits for non-polymeric compounds). Limiting the search to non-polymeric tri­phenyl­amines with the same aromatic ring at the three *para*-positions, there were 19 hits (16 compounds), which included eleven hits (nine compounds) with heteroaromatic rings and eight hits (seven compounds) with phenyl rings. The seven compounds with phenyl rings at the three *para*-position of the tri­phenyl­amine skeleton include tris­(biphenyl-4-yl)amine [WEHLIE (Inada *et al.*, 1994 ▸); WEHLIE01 (Nieger *et al.*, 2017 ▸)] and its radical cation perchlorate salt (BPHAMP10; Brown *et al.*, 1977 ▸), tris­[4-(2,3,4,5,6-penta­phenyl­phen­yl)phen­yl]amine (PULSAR; Gagnon *et al.*, 2010 ▸), tri[4-(4-formyl­phen­yl)phen­yl]amine (DEHYUN; Fang *et al.*, 2017 ▸), tri[4-(3-formyl­phen­yl)phen­yl]amine (MADJAF; Mondal *et al.*, 2016 ▸), tris­[4′-(4,6-di­amino­triazin-2-yl)biphenyl-4-yl]amine (MUNNER; Feng *et al.*, 2020 ▸) and tri[4-(4-meth­oxy­carbonyl­phen­yl)phen­yl]amine (XAXKIT; Zhang *et al.*, 2017 ▸). It is notable that there is only one reported example where the three polycyclic aromatic groups on the periphery of the tripenyl­amine skeleton are the same, *viz*. tris­[4-(quinolin-2-yl)phen­yl]amine (BEFCEX; Hariharan *et al.*, 2016 ▸).

## Synthesis and crystallization   

The title compound was prepared by a modification of the previously reported Suzuki–Miyaura coupling reaction (Kwon *et al.*, 2010 ▸). Tris(4-bromo­phen­yl)amine (2.00 g, 4.15 mmol), 1-naphthyl­boronic acid (3.57 g, 20.7 mmol), tetra­kis­(tri­phenyl­phosphine)palladium(0) (240 mg, 0.21 mmol), K_2_CO_3_ (2.87 g, 20.7 mmol), toluene (42 mL) and water (10.4 mL) were placed in a 100 mL round-bottom flask. After the solution was purged with nitro­gen for 10 minutes, it was heated at 373 K under nitro­gen for 24 h. The reaction mixture was extracted with ethyl acetate. After drying over anhydrous Na_2_SO_4_, the organic layer was evaporated. The residue was redissolved in a small amount of ethyl acetate. The addition of a large amount of methanol gave the pure product as a white precipitate (845 mg, 1.35 mmol, 33%). Colorless single crystals suitable for X-ray diffraction were obtained by means of the vapor diffusion method from chloro­form as a rich solvent and *n*-hexane as a poor solvent after standing for one week.

## Refinement   

Crystal data, data collection and structure refinement details are summarized in Table 2 ▸. C-bound H atoms were placed in geometrically calculated positions (C—H = 0.95 Å) and refined using a riding model with *U*_iso_(H) = 1.2*U*_eq_(C). One outlier (011) was omitted from the refinement.

## Supplementary Material

Crystal structure: contains datablock(s) global, I. DOI: 10.1107/S2056989020012529/is5556sup1.cif

Structure factors: contains datablock(s) I. DOI: 10.1107/S2056989020012529/is5556Isup2.hkl

Supporting information file. DOI: 10.1107/S2056989020012529/is5556Isup3.cml

CCDC reference: 2031765

Additional supporting information:  crystallographic information; 3D view; checkCIF report

## Figures and Tables

**Table 1:** Hydrogen-bond geometry (Å, °) *Cg*1, *Cg*2 and *Cg*3 are the centroids of the rings C24–C28/C33, C2–C7 and C12–C17, respectively.

*D*—H⋯*A*	*D*—H	H⋯*A*	*D*⋯*A*	*D*—H⋯*A*
C9—H9⋯*Cg*1^i^	0.95	2.65	3.5309 (19)	154
C20—H20⋯*Cg*2^i^	0.95	2.91	3.8029 (19)	156
C23—H23⋯*Cg*2^ii^	0.95	2.71	3.6165 (18)	159
C47—H47⋯*Cg*3^ii^	0.95	2.99	3.660 (2)	129

Symmetry codes: (i) 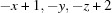; (ii) 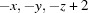.

**Table 2:** Experimental details

Crystal data	
Chemical formula	C_48_H_33_N
*M*_r_	623.80
Crystal system, space group	Triclinic, *P* 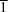
Temperature (K)	173
*a*, *b*, *c* (Å)	10.0952 (4), 13.0135 (6), 13.5643 (5)
α, β, γ (°)	74.429 (5), 75.671 (5), 78.309 (6)
*V* (Å^3^)	1645.39 (13)
*Z*	2
Radiation type	Mo *K*α
μ (mm^−1^)	0.07
Crystal size (mm)	0.50 × 0.40 × 0.25

Data collection	
Diffractometer	Rigaku R-AXIS RAPID
Absorption correction	Multi-scan (*ABSCOR*; Higashi, 1995 ▸)
*T*_min_, *T*_max_	0.689, 0.982
No. of measured, independent and observed [*F* ^2^ > 2.0σ(*F* ^2^)] reflections	16018, 7480, 5597
*R*_int_	0.030
(sin θ/λ)_max_ (Å^−1^)	0.649

Refinement	
*R*[*F* ^2^ > 2σ(*F* ^2^)], *wR*(*F* ^2^), *S*	0.053, 0.123, 1.04
No. of reflections	7480
No. of parameters	442
H-atom treatment	H-atom parameters constrained
Δρ_max_, Δρ_min_ (e Å^−3^)	0.44, −0.19

Computer programs: *RAPID-AUTO* (Rigaku, 2006 ▸), *SIR92* (Altomare, *et al.*, 1994 ▸), *SHELXL2014/7* (Sheldrick, 2015 ▸), *PLATON* (Spek, 2020 ▸), *publCIF* (Westrip, 2010 ▸) and *CrystalStructure* (Rigaku, 2016 ▸).

## supplementary crystallographic information

### Crystal data

**Table:**

C_48_H_33_N	*Z* = 2
*M**_r_* = 623.80	*F*(000) = 656.00
Triclinic, *P*1	*D*_x_ = 1.259 Mg m^−^^3^
*a* = 10.0952 (4) Å	Mo *K*α radiation, λ = 0.71075 Å
*b* = 13.0135 (6) Å	Cell parameters from 12529 reflections
*c* = 13.5643 (5) Å	θ = 2.1–27.5°
α = 74.429 (5)°	µ = 0.07 mm^−^^1^
β = 75.671 (5)°	*T* = 173 K
γ = 78.309 (6)°	Block, colorless
*V* = 1645.39 (13) Å^3^	0.50 × 0.40 × 0.25 mm

### Data collection

**Table:**

Rigaku R-AXIS RAPID diffractometer	5597 reflections with *F*^2^ > 2.0σ(*F*^2^)
Detector resolution: 10.000 pixels mm^-1^	*R*_int_ = 0.030
ω scans	θ_max_ = 27.5°, θ_min_ = 2.1°
Absorption correction: multi-scan (*ABSCOR*; Higashi, 1995)	*h* = −13→13
*T*_min_ = 0.689, *T*_max_ = 0.982	*k* = −16→16
16018 measured reflections	*l* = −17→17
7480 independent reflections	

### Refinement

**Table:**

Refinement on *F*^2^	Secondary atom site location: difference Fourier map
*R*[*F*^2^ > 2σ(*F*^2^)] = 0.053	Hydrogen site location: inferred from neighbouring sites
*wR*(*F*^2^) = 0.123	H-atom parameters constrained
*S* = 1.04	*w* = 1/[σ^2^(*F*_o_^2^) + (0.0463*P*)^2^ + 0.6997*P*] where *P* = (*F*_o_^2^ + 2*F*_c_^2^)/3
7480 reflections	(Δ/σ)_max_ < 0.001
442 parameters	Δρ_max_ = 0.44 e Å^−^^3^
0 restraints	Δρ_min_ = −0.19 e Å^−^^3^
Primary atom site location: structure-invariant direct methods	

### Special details

**Table:**

Geometry. All esds (except the esd in the dihedral angle between two l.s. planes) are estimated using the full covariance matrix. The cell esds are taken into account individually in the estimation of esds in distances, angles and torsion angles; correlations between esds in cell parameters are only used when they are defined by crystal symmetry. An approximate (isotropic) treatment of cell esds is used for estimating esds involving l.s. planes.
Refinement. Refinement was performed using all reflections. The weighted R-factor (wR) and goodness of fit (S) are based on F^2^. R-factor (gt) are based on F. The threshold expression of F^2^ > 2.0 sigma(F^2^) is used only for calculating R-factor (gt).

### Fractional atomic coordinates and isotropic or equivalent isotropic displacement parameters (Å^2^)

**Table:**

	*x*	*y*	*z*	*U*_iso_*/*U*_eq_	
N1	0.14801 (14)	0.09044 (10)	1.02472 (10)	0.0266 (3)	
C2	0.16313 (15)	−0.00593 (12)	1.10268 (12)	0.0232 (3)	
C3	0.13832 (16)	−0.00538 (12)	1.20859 (12)	0.0259 (3)	
H3	0.1124	0.0612	1.2295	0.031*	
C4	0.15124 (16)	−0.10144 (12)	1.28352 (12)	0.0260 (3)	
H4	0.1334	−0.0992	1.3551	0.031*	
C5	0.18977 (15)	−0.20126 (12)	1.25653 (12)	0.0240 (3)	
C6	0.21653 (16)	−0.20035 (12)	1.15008 (12)	0.0248 (3)	
H6	0.2436	−0.2668	1.1291	0.030*	
C7	0.20467 (15)	−0.10512 (12)	1.07433 (12)	0.0248 (3)	
H7	0.2249	−0.1073	1.0026	0.030*	
C8	0.21175 (16)	−0.30334 (12)	1.33678 (12)	0.0259 (3)	
C9	0.29701 (17)	−0.30985 (13)	1.40386 (13)	0.0311 (4)	
H9	0.3343	−0.2476	1.4016	0.037*	
C10	0.33033 (19)	−0.40671 (14)	1.47592 (14)	0.0357 (4)	
H10	0.3897	−0.4092	1.5212	0.043*	
C11	0.27776 (19)	−0.49663 (14)	1.48081 (14)	0.0358 (4)	
H11	0.3029	−0.5620	1.5283	0.043*	
C12	0.18616 (18)	−0.49406 (13)	1.41617 (13)	0.0305 (4)	
C13	0.12495 (19)	−0.58567 (14)	1.42374 (15)	0.0371 (4)	
H13	0.1493	−0.6515	1.4709	0.045*	
C14	0.0320 (2)	−0.58075 (15)	1.36455 (15)	0.0413 (4)	
H14	−0.0071	−0.6431	1.3700	0.050*	
C15	−0.00617 (19)	−0.48328 (15)	1.29518 (15)	0.0386 (4)	
H15	−0.0724	−0.4799	1.2550	0.046*	
C16	0.05106 (17)	−0.39365 (14)	1.28512 (13)	0.0316 (4)	
H16	0.0241	−0.3287	1.2378	0.038*	
C17	0.15007 (16)	−0.39574 (13)	1.34394 (12)	0.0269 (3)	
C18	0.23208 (16)	0.09424 (11)	0.92230 (12)	0.0242 (3)	
C19	0.37499 (17)	0.07939 (12)	0.90874 (13)	0.0281 (3)	
H19	0.4165	0.0695	0.9669	0.034*	
C20	0.45771 (17)	0.07890 (13)	0.81001 (13)	0.0291 (4)	
H20	0.5554	0.0670	0.8019	0.035*	
C21	0.39922 (17)	0.09568 (12)	0.72244 (13)	0.0275 (3)	
C22	0.25551 (17)	0.11267 (13)	0.73763 (13)	0.0296 (4)	
H22	0.2133	0.1256	0.6793	0.036*	
C23	0.17291 (16)	0.11113 (13)	0.83621 (13)	0.0278 (3)	
H23	0.0752	0.1217	0.8448	0.033*	
C24	0.48684 (17)	0.10367 (13)	0.61483 (13)	0.0291 (4)	
C25	0.45260 (18)	0.18906 (13)	0.53581 (13)	0.0321 (4)	
H25	0.3686	0.2363	0.5493	0.039*	
C26	0.5382 (2)	0.20880 (16)	0.43537 (14)	0.0430 (5)	
H26	0.5112	0.2679	0.3821	0.052*	
C27	0.6607 (2)	0.14220 (16)	0.41495 (14)	0.0415 (4)	
H27	0.7210	0.1574	0.3485	0.050*	
C28	0.69744 (18)	0.05122 (14)	0.49235 (13)	0.0330 (4)	
C29	0.8225 (2)	−0.02024 (16)	0.47202 (15)	0.0416 (4)	
H29	0.8847	−0.0036	0.4067	0.050*	
C30	0.8555 (2)	−0.11158 (16)	0.54362 (16)	0.0423 (5)	
H30	0.9398	−0.1580	0.5286	0.051*	
C31	0.76354 (19)	−0.13658 (15)	0.64002 (15)	0.0381 (4)	
H31	0.7841	−0.2022	0.6887	0.046*	
C32	0.64506 (18)	−0.06841 (13)	0.66525 (13)	0.0307 (4)	
H32	0.5862	−0.0861	0.7320	0.037*	
C33	0.60871 (17)	0.02880 (13)	0.59287 (13)	0.0297 (4)	
C34	0.04483 (15)	0.17917 (12)	1.04198 (12)	0.0236 (3)	
C35	0.06416 (16)	0.28295 (12)	0.98293 (12)	0.0265 (3)	
H35	0.1471	0.2937	0.9324	0.032*	
C36	−0.03641 (17)	0.37021 (12)	0.99736 (13)	0.0275 (3)	
H36	−0.0224	0.4398	0.9551	0.033*	
C37	−0.15856 (16)	0.35813 (12)	1.07296 (12)	0.0257 (3)	
C38	−0.17683 (16)	0.25404 (12)	1.13011 (12)	0.0264 (3)	
H38	−0.2595	0.2433	1.1809	0.032*	
C39	−0.07832 (16)	0.16580 (12)	1.11521 (12)	0.0256 (3)	
H39	−0.0946	0.0957	1.1550	0.031*	
C40	−0.26129 (16)	0.45250 (12)	1.09758 (13)	0.0270 (3)	
C41	−0.29800 (18)	0.46387 (14)	1.19948 (14)	0.0330 (4)	
H41	−0.2614	0.4097	1.2524	0.040*	
C42	−0.38881 (19)	0.55428 (15)	1.22681 (15)	0.0376 (4)	
H42	−0.4133	0.5601	1.2976	0.045*	
C43	−0.44133 (18)	0.63313 (14)	1.15188 (15)	0.0358 (4)	
H43	−0.4994	0.6951	1.1705	0.043*	
C44	−0.41071 (16)	0.62406 (12)	1.04683 (14)	0.0298 (4)	
C45	−0.46871 (18)	0.70263 (14)	0.96833 (15)	0.0372 (4)	
H45	−0.5255	0.7657	0.9854	0.045*	
C46	−0.44480 (19)	0.68960 (14)	0.86875 (15)	0.0398 (4)	
H46	−0.4853	0.7432	0.8174	0.048*	
C47	−0.36022 (19)	0.59692 (15)	0.84179 (14)	0.0376 (4)	
H47	−0.3453	0.5874	0.7727	0.045*	
C48	−0.29959 (17)	0.52080 (13)	0.91465 (13)	0.0314 (4)	
H48	−0.2416	0.4592	0.8951	0.038*	
C49	−0.32131 (16)	0.53158 (12)	1.01890 (13)	0.0264 (3)	

### Atomic displacement parameters (Å^2^)

**Table:**

	*U*^11^	*U*^22^	*U*^33^	*U*^12^	*U*^13^	*U*^23^
N1	0.0254 (7)	0.0233 (6)	0.0238 (7)	0.0055 (5)	−0.0008 (5)	−0.0039 (5)
C2	0.0173 (7)	0.0223 (7)	0.0265 (8)	0.0025 (6)	−0.0038 (6)	−0.0045 (6)
C3	0.0246 (8)	0.0248 (7)	0.0278 (8)	0.0040 (6)	−0.0067 (6)	−0.0094 (7)
C4	0.0226 (8)	0.0300 (8)	0.0245 (8)	0.0012 (6)	−0.0045 (6)	−0.0089 (7)
C5	0.0189 (7)	0.0241 (7)	0.0267 (8)	−0.0006 (6)	−0.0044 (6)	−0.0044 (6)
C6	0.0221 (8)	0.0218 (7)	0.0295 (8)	0.0011 (6)	−0.0037 (6)	−0.0088 (6)
C7	0.0207 (8)	0.0273 (8)	0.0249 (8)	0.0016 (6)	−0.0034 (6)	−0.0086 (6)
C8	0.0230 (8)	0.0259 (8)	0.0251 (8)	0.0009 (6)	−0.0020 (6)	−0.0059 (6)
C9	0.0313 (9)	0.0282 (8)	0.0326 (9)	−0.0022 (7)	−0.0089 (7)	−0.0044 (7)
C10	0.0348 (10)	0.0369 (9)	0.0336 (9)	0.0018 (7)	−0.0142 (8)	−0.0040 (8)
C11	0.0374 (10)	0.0282 (8)	0.0344 (9)	0.0032 (7)	−0.0084 (8)	0.0004 (7)
C12	0.0311 (9)	0.0267 (8)	0.0284 (8)	0.0009 (6)	−0.0001 (7)	−0.0066 (7)
C13	0.0388 (10)	0.0272 (8)	0.0387 (10)	−0.0022 (7)	−0.0010 (8)	−0.0049 (8)
C14	0.0409 (11)	0.0342 (9)	0.0480 (11)	−0.0125 (8)	0.0020 (9)	−0.0132 (9)
C15	0.0339 (10)	0.0446 (10)	0.0394 (10)	−0.0102 (8)	−0.0057 (8)	−0.0116 (9)
C16	0.0278 (9)	0.0338 (9)	0.0305 (9)	−0.0041 (7)	−0.0028 (7)	−0.0060 (7)
C17	0.0240 (8)	0.0276 (8)	0.0252 (8)	−0.0001 (6)	0.0002 (6)	−0.0070 (7)
C18	0.0234 (8)	0.0184 (7)	0.0266 (8)	0.0018 (6)	−0.0006 (6)	−0.0057 (6)
C19	0.0260 (8)	0.0273 (8)	0.0283 (8)	0.0021 (6)	−0.0069 (7)	−0.0050 (7)
C20	0.0208 (8)	0.0284 (8)	0.0348 (9)	0.0005 (6)	−0.0019 (6)	−0.0081 (7)
C21	0.0283 (8)	0.0236 (7)	0.0288 (8)	−0.0029 (6)	−0.0013 (7)	−0.0078 (7)
C22	0.0283 (9)	0.0318 (8)	0.0294 (8)	−0.0035 (7)	−0.0065 (7)	−0.0084 (7)
C23	0.0205 (8)	0.0295 (8)	0.0320 (9)	−0.0011 (6)	−0.0028 (6)	−0.0088 (7)
C24	0.0309 (9)	0.0284 (8)	0.0291 (8)	−0.0077 (7)	−0.0050 (7)	−0.0068 (7)
C25	0.0335 (9)	0.0310 (8)	0.0309 (9)	−0.0045 (7)	−0.0072 (7)	−0.0048 (7)
C26	0.0542 (12)	0.0436 (10)	0.0297 (9)	−0.0134 (9)	−0.0123 (9)	0.0021 (8)
C27	0.0475 (12)	0.0501 (11)	0.0246 (9)	−0.0151 (9)	−0.0010 (8)	−0.0046 (8)
C28	0.0348 (10)	0.0396 (9)	0.0274 (8)	−0.0123 (7)	−0.0004 (7)	−0.0128 (8)
C29	0.0372 (11)	0.0522 (11)	0.0377 (10)	−0.0112 (9)	0.0036 (8)	−0.0210 (9)
C30	0.0321 (10)	0.0448 (11)	0.0517 (12)	−0.0020 (8)	0.0002 (8)	−0.0250 (10)
C31	0.0381 (10)	0.0336 (9)	0.0439 (10)	−0.0026 (7)	−0.0057 (8)	−0.0155 (8)
C32	0.0321 (9)	0.0269 (8)	0.0318 (9)	−0.0053 (7)	−0.0017 (7)	−0.0081 (7)
C33	0.0304 (9)	0.0315 (8)	0.0287 (8)	−0.0088 (7)	−0.0016 (7)	−0.0104 (7)
C34	0.0210 (8)	0.0227 (7)	0.0255 (8)	0.0038 (6)	−0.0050 (6)	−0.0076 (6)
C35	0.0223 (8)	0.0261 (8)	0.0285 (8)	−0.0016 (6)	0.0000 (6)	−0.0081 (7)
C36	0.0280 (8)	0.0208 (7)	0.0314 (8)	−0.0021 (6)	−0.0031 (7)	−0.0058 (7)
C37	0.0243 (8)	0.0247 (7)	0.0271 (8)	0.0040 (6)	−0.0060 (6)	−0.0092 (6)
C38	0.0208 (8)	0.0286 (8)	0.0252 (8)	0.0020 (6)	−0.0017 (6)	−0.0055 (7)
C39	0.0251 (8)	0.0219 (7)	0.0262 (8)	0.0013 (6)	−0.0054 (6)	−0.0028 (6)
C40	0.0226 (8)	0.0243 (7)	0.0323 (9)	0.0020 (6)	−0.0034 (6)	−0.0096 (7)
C41	0.0295 (9)	0.0340 (9)	0.0332 (9)	0.0047 (7)	−0.0065 (7)	−0.0109 (7)
C42	0.0333 (10)	0.0438 (10)	0.0365 (10)	0.0040 (8)	−0.0034 (8)	−0.0212 (8)
C43	0.0266 (9)	0.0318 (9)	0.0481 (11)	0.0052 (7)	−0.0023 (8)	−0.0199 (8)
C44	0.0206 (8)	0.0247 (8)	0.0412 (10)	0.0000 (6)	−0.0018 (7)	−0.0094 (7)
C45	0.0274 (9)	0.0256 (8)	0.0499 (11)	0.0035 (7)	−0.0020 (8)	−0.0046 (8)
C46	0.0298 (9)	0.0335 (9)	0.0434 (11)	0.0003 (7)	−0.0063 (8)	0.0077 (8)
C47	0.0336 (10)	0.0413 (10)	0.0320 (9)	−0.0039 (8)	−0.0035 (7)	−0.0024 (8)
C48	0.0285 (9)	0.0288 (8)	0.0331 (9)	0.0007 (7)	−0.0027 (7)	−0.0075 (7)
C49	0.0207 (8)	0.0233 (7)	0.0324 (9)	−0.0009 (6)	−0.0017 (6)	−0.0070 (7)

### Geometric parameters (Å, º)

**Table:**

N1—C2	1.4159 (19)	C25—C26	1.410 (2)
N1—C34	1.4179 (18)	C25—H25	0.9500
N1—C18	1.4315 (19)	C26—C27	1.372 (3)
C2—C3	1.398 (2)	C26—H26	0.9500
C2—C7	1.399 (2)	C27—C28	1.410 (3)
C3—C4	1.389 (2)	C27—H27	0.9500
C3—H3	0.9500	C28—C29	1.423 (3)
C4—C5	1.397 (2)	C28—C33	1.426 (2)
C4—H4	0.9500	C29—C30	1.359 (3)
C5—C6	1.399 (2)	C29—H29	0.9500
C5—C8	1.491 (2)	C30—C31	1.404 (3)
C6—C7	1.387 (2)	C30—H30	0.9500
C6—H6	0.9500	C31—C32	1.366 (2)
C7—H7	0.9500	C31—H31	0.9500
C8—C9	1.376 (2)	C32—C33	1.422 (2)
C8—C17	1.433 (2)	C32—H32	0.9500
C9—C10	1.410 (2)	C34—C39	1.395 (2)
C9—H9	0.9500	C34—C35	1.397 (2)
C10—C11	1.359 (3)	C35—C36	1.383 (2)
C10—H10	0.9500	C35—H35	0.9500
C11—C12	1.414 (3)	C36—C37	1.401 (2)
C11—H11	0.9500	C36—H36	0.9500
C12—C13	1.419 (2)	C37—C38	1.392 (2)
C12—C17	1.430 (2)	C37—C40	1.493 (2)
C13—C14	1.360 (3)	C38—C39	1.382 (2)
C13—H13	0.9500	C38—H38	0.9500
C14—C15	1.408 (3)	C39—H39	0.9500
C14—H14	0.9500	C40—C41	1.380 (2)
C15—C16	1.364 (3)	C40—C49	1.429 (2)
C15—H15	0.9500	C41—C42	1.411 (2)
C16—C17	1.417 (2)	C41—H41	0.9500
C16—H16	0.9500	C42—C43	1.361 (3)
C18—C23	1.387 (2)	C42—H42	0.9500
C18—C19	1.388 (2)	C43—C44	1.413 (3)
C19—C20	1.393 (2)	C43—H43	0.9500
C19—H19	0.9500	C44—C45	1.415 (2)
C20—C21	1.401 (2)	C44—C49	1.429 (2)
C20—H20	0.9500	C45—C46	1.363 (3)
C21—C22	1.394 (2)	C45—H45	0.9500
C21—C24	1.497 (2)	C46—C47	1.408 (3)
C22—C23	1.387 (2)	C46—H46	0.9500
C22—H22	0.9500	C47—C48	1.364 (2)
C23—H23	0.9500	C47—H47	0.9500
C24—C25	1.375 (2)	C48—C49	1.417 (2)
C24—C33	1.428 (2)	C48—H48	0.9500
			
C2—N1—C34	122.34 (13)	C26—C25—H25	118.9
C2—N1—C18	118.41 (12)	C27—C26—C25	119.68 (17)
C34—N1—C18	119.01 (12)	C27—C26—H26	120.2
C3—C2—C7	118.28 (14)	C25—C26—H26	120.2
C3—C2—N1	121.81 (14)	C26—C27—C28	120.15 (17)
C7—C2—N1	119.91 (14)	C26—C27—H27	119.9
C4—C3—C2	120.50 (14)	C28—C27—H27	119.9
C4—C3—H3	119.8	C27—C28—C29	121.05 (16)
C2—C3—H3	119.8	C27—C28—C33	120.29 (16)
C3—C4—C5	121.84 (15)	C29—C28—C33	118.65 (16)
C3—C4—H4	119.1	C30—C29—C28	121.74 (17)
C5—C4—H4	119.1	C30—C29—H29	119.1
C4—C5—C6	116.98 (14)	C28—C29—H29	119.1
C4—C5—C8	121.42 (14)	C29—C30—C31	119.25 (18)
C6—C5—C8	121.44 (14)	C29—C30—H30	120.4
C7—C6—C5	121.86 (14)	C31—C30—H30	120.4
C7—C6—H6	119.1	C32—C31—C30	121.33 (18)
C5—C6—H6	119.1	C32—C31—H31	119.3
C6—C7—C2	120.52 (14)	C30—C31—H31	119.3
C6—C7—H7	119.7	C31—C32—C33	120.81 (16)
C2—C7—H7	119.7	C31—C32—H32	119.6
C9—C8—C17	119.14 (14)	C33—C32—H32	119.6
C9—C8—C5	118.90 (15)	C32—C33—C28	118.03 (15)
C17—C8—C5	121.93 (14)	C32—C33—C24	123.46 (15)
C8—C9—C10	121.53 (16)	C28—C33—C24	118.49 (15)
C8—C9—H9	119.2	C39—C34—C35	118.44 (13)
C10—C9—H9	119.2	C39—C34—N1	121.76 (14)
C11—C10—C9	120.23 (17)	C35—C34—N1	119.78 (14)
C11—C10—H10	119.9	C36—C35—C34	120.59 (14)
C9—C10—H10	119.9	C36—C35—H35	119.7
C10—C11—C12	120.84 (16)	C34—C35—H35	119.7
C10—C11—H11	119.6	C35—C36—C37	121.41 (14)
C12—C11—H11	119.6	C35—C36—H36	119.3
C11—C12—C13	121.65 (16)	C37—C36—H36	119.3
C11—C12—C17	119.32 (15)	C38—C37—C36	117.18 (14)
C13—C12—C17	118.99 (16)	C38—C37—C40	120.52 (14)
C14—C13—C12	121.10 (17)	C36—C37—C40	122.16 (14)
C14—C13—H13	119.4	C39—C38—C37	122.02 (14)
C12—C13—H13	119.4	C39—C38—H38	119.0
C13—C14—C15	120.00 (17)	C37—C38—H38	119.0
C13—C14—H14	120.0	C38—C39—C34	120.31 (14)
C15—C14—H14	120.0	C38—C39—H39	119.8
C16—C15—C14	120.66 (18)	C34—C39—H39	119.8
C16—C15—H15	119.7	C41—C40—C49	119.25 (14)
C14—C15—H15	119.7	C41—C40—C37	118.67 (14)
C15—C16—C17	121.18 (16)	C49—C40—C37	122.08 (14)
C15—C16—H16	119.4	C40—C41—C42	121.39 (16)
C17—C16—H16	119.4	C40—C41—H41	119.3
C16—C17—C12	118.04 (15)	C42—C41—H41	119.3
C16—C17—C8	123.11 (15)	C43—C42—C41	120.07 (17)
C12—C17—C8	118.82 (15)	C43—C42—H42	120.0
C23—C18—C19	119.28 (14)	C41—C42—H42	120.0
C23—C18—N1	120.95 (14)	C42—C43—C44	120.94 (15)
C19—C18—N1	119.77 (14)	C42—C43—H43	119.5
C18—C19—C20	120.17 (15)	C44—C43—H43	119.5
C18—C19—H19	119.9	C43—C44—C45	122.04 (15)
C20—C19—H19	119.9	C43—C44—C49	119.32 (15)
C19—C20—C21	121.09 (15)	C45—C44—C49	118.62 (16)
C19—C20—H20	119.5	C46—C45—C44	121.35 (16)
C21—C20—H20	119.5	C46—C45—H45	119.3
C22—C21—C20	117.70 (15)	C44—C45—H45	119.3
C22—C21—C24	120.65 (15)	C45—C46—C47	120.13 (17)
C20—C21—C24	121.49 (15)	C45—C46—H46	119.9
C23—C22—C21	121.31 (16)	C47—C46—H46	119.9
C23—C22—H22	119.3	C48—C47—C46	120.19 (18)
C21—C22—H22	119.3	C48—C47—H47	119.9
C18—C23—C22	120.43 (15)	C46—C47—H47	119.9
C18—C23—H23	119.8	C47—C48—C49	121.42 (16)
C22—C23—H23	119.8	C47—C48—H48	119.3
C25—C24—C33	119.01 (15)	C49—C48—H48	119.3
C25—C24—C21	118.78 (15)	C48—C49—C44	118.24 (15)
C33—C24—C21	122.12 (14)	C48—C49—C40	122.85 (14)
C24—C25—C26	122.10 (17)	C44—C49—C40	118.88 (15)
C24—C25—H25	118.9		
			
C34—N1—C2—C3	−35.0 (2)	C24—C25—C26—C27	−0.8 (3)
C18—N1—C2—C3	150.63 (15)	C25—C26—C27—C28	3.3 (3)
C34—N1—C2—C7	145.32 (15)	C26—C27—C28—C29	178.57 (18)
C18—N1—C2—C7	−29.0 (2)	C26—C27—C28—C33	−1.1 (3)
C7—C2—C3—C4	−1.7 (2)	C27—C28—C29—C30	−176.10 (19)
N1—C2—C3—C4	178.66 (14)	C33—C28—C29—C30	3.5 (3)
C2—C3—C4—C5	0.4 (2)	C28—C29—C30—C31	0.3 (3)
C3—C4—C5—C6	0.7 (2)	C29—C30—C31—C32	−3.2 (3)
C3—C4—C5—C8	176.20 (15)	C30—C31—C32—C33	2.1 (3)
C4—C5—C6—C7	−0.4 (2)	C31—C32—C33—C28	1.8 (3)
C8—C5—C6—C7	−175.91 (14)	C31—C32—C33—C24	−179.57 (17)
C5—C6—C7—C2	−0.9 (2)	C27—C28—C33—C32	175.11 (17)
C3—C2—C7—C6	2.0 (2)	C29—C28—C33—C32	−4.5 (2)
N1—C2—C7—C6	−178.37 (14)	C27—C28—C33—C24	−3.6 (2)
C4—C5—C8—C9	−51.0 (2)	C29—C28—C33—C24	176.78 (16)
C6—C5—C8—C9	124.33 (17)	C25—C24—C33—C32	−172.66 (16)
C4—C5—C8—C17	130.98 (17)	C21—C24—C33—C32	10.7 (3)
C6—C5—C8—C17	−53.7 (2)	C25—C24—C33—C28	6.0 (2)
C17—C8—C9—C10	3.1 (2)	C21—C24—C33—C28	−170.65 (15)
C5—C8—C9—C10	−175.02 (15)	C2—N1—C34—C39	−26.1 (2)
C8—C9—C10—C11	−0.2 (3)	C18—N1—C34—C39	148.20 (15)
C9—C10—C11—C12	−1.8 (3)	C2—N1—C34—C35	155.43 (15)
C10—C11—C12—C13	−176.88 (17)	C18—N1—C34—C35	−30.3 (2)
C10—C11—C12—C17	0.8 (3)	C39—C34—C35—C36	0.3 (2)
C11—C12—C13—C14	176.91 (17)	N1—C34—C35—C36	178.83 (15)
C17—C12—C13—C14	−0.8 (3)	C34—C35—C36—C37	1.7 (3)
C12—C13—C14—C15	−0.7 (3)	C35—C36—C37—C38	−2.5 (2)
C13—C14—C15—C16	1.2 (3)	C35—C36—C37—C40	173.31 (15)
C14—C15—C16—C17	−0.1 (3)	C36—C37—C38—C39	1.3 (2)
C15—C16—C17—C12	−1.4 (2)	C40—C37—C38—C39	−174.58 (16)
C15—C16—C17—C8	−179.29 (16)	C37—C38—C39—C34	0.7 (2)
C11—C12—C17—C16	−175.91 (15)	C35—C34—C39—C38	−1.5 (2)
C13—C12—C17—C16	1.8 (2)	N1—C34—C39—C38	−179.98 (15)
C11—C12—C17—C8	2.1 (2)	C38—C37—C40—C41	51.8 (2)
C13—C12—C17—C8	179.79 (15)	C36—C37—C40—C41	−123.81 (18)
C9—C8—C17—C16	173.91 (15)	C38—C37—C40—C49	−128.84 (17)
C5—C8—C17—C16	−8.0 (2)	C36—C37—C40—C49	55.5 (2)
C9—C8—C17—C12	−4.0 (2)	C49—C40—C41—C42	−2.8 (3)
C5—C8—C17—C12	174.10 (14)	C37—C40—C41—C42	176.55 (16)
C2—N1—C18—C23	118.54 (16)	C40—C41—C42—C43	−0.6 (3)
C34—N1—C18—C23	−56.0 (2)	C41—C42—C43—C44	2.6 (3)
C2—N1—C18—C19	−60.6 (2)	C42—C43—C44—C45	177.23 (17)
C34—N1—C18—C19	124.90 (16)	C42—C43—C44—C49	−1.1 (3)
C23—C18—C19—C20	−1.6 (2)	C43—C44—C45—C46	−176.00 (17)
N1—C18—C19—C20	177.57 (14)	C49—C44—C45—C46	2.4 (3)
C18—C19—C20—C21	1.5 (2)	C44—C45—C46—C47	−0.4 (3)
C19—C20—C21—C22	−0.1 (2)	C45—C46—C47—C48	−1.3 (3)
C19—C20—C21—C24	175.28 (15)	C46—C47—C48—C49	1.0 (3)
C20—C21—C22—C23	−1.1 (2)	C47—C48—C49—C44	0.9 (2)
C24—C21—C22—C23	−176.59 (15)	C47—C48—C49—C40	178.92 (16)
C19—C18—C23—C22	0.3 (2)	C43—C44—C49—C48	175.83 (16)
N1—C18—C23—C22	−178.81 (14)	C45—C44—C49—C48	−2.6 (2)
C21—C22—C23—C18	1.1 (2)	C43—C44—C49—C40	−2.2 (2)
C22—C21—C24—C25	45.1 (2)	C45—C44—C49—C40	179.36 (15)
C20—C21—C24—C25	−130.13 (17)	C41—C40—C49—C48	−173.82 (16)
C22—C21—C24—C33	−138.26 (17)	C37—C40—C49—C48	6.8 (2)
C20—C21—C24—C33	46.5 (2)	C41—C40—C49—C44	4.1 (2)
C33—C24—C25—C26	−3.9 (3)	C37—C40—C49—C44	−175.20 (15)
C21—C24—C25—C26	172.84 (16)		

### Hydrogen-bond geometry (Å, º)

*Cg*1, *Cg*2 and *Cg*3 are the centroids of the rings C24–C28/C33, C2–C7 and C12–C17, respectively.

**Table:**

*D*—H···*A*	*D*—H	H···*A*	*D*···*A*	*D*—H···*A*
C9—H9···*Cg*1^i^	0.95	2.65	3.5309 (19)	154
C20—H20···*Cg*2^i^	0.95	2.91	3.8029 (19)	156
C23—H23···*Cg*2^ii^	0.95	2.71	3.6165 (18)	159
C47—H47···*Cg*3^ii^	0.95	2.99	3.660 (2)	129

Symmetry codes: (i) −*x*+1, −*y*, −*z*+2; (ii) −*x*, −*y*, −*z*+2.
